# The Nexus of Hyperparathyroidism and Thyroid Carcinoma: Insights into Pathogenesis and Diagnostic Challenges—A Narrative Review

**DOI:** 10.3390/jcm13010147

**Published:** 2023-12-27

**Authors:** Gregorio Scerrino, Nunzia Cinzia Paladino, Giuseppina Orlando, Giuseppe Salamone, Pierina Richiusa, Stefano Radellini, Giuseppina Melfa, Giuseppa Graceffa

**Affiliations:** 1 Unit of Endocrine Surgery, Department of Surgical Oncology and Oral Sciences, University of Palermo, 90127 Palermo, Italy; 2 Department of General Endocrine and Metabolic Surgery, Conception Hospital, Aix-Marseille University, 147, Boulevard Baille, 13005 Marseille, France; nunzia.paladino@ap-hm.fr; 3 Unit of General and Emergency Surgery, Department of Surgical Oncology and Oral Sciences, University of Palermo, 90127 Palermo, Italy; giuseppina.orlando@policlinico.pa.it (G.O.); giuseppe.salamone@unipa.it (G.S.); irene_melfa@yahoo.it (G.M.); 4 Section of Endocrinology, Department of Health Promotion Sciences Maternal and Infantile Care, Internal Medicine and Medical Specialties (PROMISE), University of Palermo, 90127 Palermo, Italy; pierina.richiusa@policlinico.pa.it (P.R.); stefano.radellini@policlinico.pa.it (S.R.); 5 Unit of General and Oncology Surgery, Department of Surgical Oncology and Oral Sciences, University of Palermo, 90127 Palermo, Italy; giuseppa.graceffa@unipa.it

**Keywords:** hyperparathyroidism, thyroid carcinoma, thyroid nodules, thyroidectomy, parathyroidectomy, PTH levels

## Abstract

This review investigates the intricate relationship between hyperparathyroidism (HPT) and thyroid carcinoma (TC), aiming to elucidate their coexistence, potential pathogenetic mechanisms, and clinical implications. A systematic search strategy, employing the MeSH terms ‘Hyperparathyroidism’ and ‘Thyroid Carcinoma’, spanned publications from 2013 to 2023 across the PubMed, Web of Science, and Scopus databases. Fifteen selected articles were analyzed. Studies unanimously confirm the notable association between primary hyperparathyroidism (PHPT) and thyroid nodules/cancer, with incidences ranging from 2.8% to 47.1%. Key findings reveal a predilection for papillary thyroid carcinoma (PTC) in this association, showcasing varying tumor characteristics and gender disparities. Lower preoperative serum parathyroid hormone (PTH) levels are a potential risk factor for thyroid cancer in PHPT patients. Diverse surgical approaches and tumor characteristics between PHPT and secondary hyperparathyroidism (SHPT) cases were noted. Moreover, this review underscores the scarcity of definitive guidelines in managing concurrent PHPT and thyroid conditions, advocating for comprehensive assessments to enhance diagnostic accuracy and refine therapeutic interventions. Rare coincidental associations, as highlighted by case reports, shed light on unique clinical scenarios. In essence, this review amalgamates evidence to deepen the understanding of the interplay between HPT and TC, emphasizing the need for further research to elucidate underlying mechanisms and guide clinical management.

## 1. Introduction

The link between hyperparathyroidism (HPT) and thyroid diseases has been a subject of growing interest in recent studies wanting to leave out the obvious association between the two conditions in the context of multiple endocrine neoplasia [1]. Its association with thyroid carcinoma in particular has been repeatedly stated [2,3]. While a significant association between these conditions has been established, several aspects remain elusive and require nuanced exploration in the current literature [4]. Notably, the precise mechanistic interplay between HPT and TC, whether causative or coincidental, merits deeper investigation. Understanding the molecular pathways or shared genetic factors contributing to their co-occurrence remains an open area for exploration [5,6]. Additionally, there is a need for comprehensive studies elucidating the impact of HPT on the prognosis, progression, or treatment outcomes of TC, and vice versa [7,8]. Clarifying the clinical implications of their concurrent presence, including diagnostic challenges and therapeutic management, stands as another pivotal aspect requiring further scholarly attention [3,8,9,10]. Furthermore, exploring potential risk factors or predisposing conditions that may underlie their coexistence would contribute substantially to a holistic understanding of this association [7]. In essence, while acknowledging the established connection between HPT and TC, ongoing research endeavors seek to unveil the underlying mechanisms, clinical implications, and therapeutic considerations, thereby refining our comprehension of this intriguing interrelationship.

The purpose of this review is to assess, in light of the most up-to-date literature, whether the coexistence of the two diseases is occasional or real and whether there are possible pathogenetic mechanisms of interrelation. It will also assess whether the form of HPT most frequently related to TC is primary (PHPT) or secondary (SHPT) and which form of TC is most frequently found in this association. Finally, issues of diagnosis and treatment will be evaluated.

## 2. Methods

The MeSH terms ‘Hyperparathyroidism’ and ‘Thyroid Carcinoma’ were utilized to curate publications encompassing a 10-year interval (2013–2023). Two independent researchers conducted searches across PubMed, Web of Science, and Scopus, systematically filtering out duplicates. Only articles published in English were considered. The data extraction encompassed publication year, type, MeSH terms, and abstract content.

Reducing the literature search to 2013–2023 aimed to focus on contemporary research encompassing the most recent advancements in the evolving landscape of hyperparathyroidism and thyroid carcinoma. This timeframe ensured a comprehensive review of the latest evidence-based findings while excluding older studies that might lack relevance to the current state of understanding.

Abstracts from the retrieved publications underwent examination. Subsequently, articles deemed relevant underwent scrutiny, involving an assessment of the original arguments. This approach facilitated the identification of diverse perspectives, novel correlations, and distinctive contributions across the literature.

The synthesis subsequently performed aimed to elucidate converging themes and discrepancies, providing a comprehensive understanding of the relationship between hyperparathyroidism and thyroid carcinoma.

## 3. Results

A total of 15 original articles and 6 case reports were deemed suitable for the present review study.

A study from Lehwald and coll. (2013) [11] investigated the coexistence of nonmedullary thyroid carcinoma (NMTC) and primary hyperparathyroidism (PHPT), aiming to understand the clinical incidence and tumor characteristics in patients with this combined pathology.

Incidence of NMTC

The research comprises a cohort of 1464 patients with PHPT over 26 years, among whom 41 patients (2.8%) also had NMTC.

NMTC variants and demographic characteristics

The majority of NMTC cases were papillary thyroid carcinoma (PTC), while a smaller percentage presented as follicular thyroid carcinoma (FTC).

Key findings included a female predominance among affected patients and a median age at operation of 58.1 years. Interestingly, patients with FTC showed significantly lower preoperative parathyroid hormone (PTH) levels compared to those with PTC.

Tumor characteristics revealed differences between PTC and FTC. PTC patients tended to have smaller tumors (mostly from 0.05–1 cm) and fewer cases of extrathyroidal invasion compared to FTC patients, who presented larger tumors (>2 cm) with increased extrathyroidal growth and more frequent lymph node metastases.

Surgical procedures

Surgical procedures differed between PTC and FTC patients, with more FTC patients undergoing thyroidectomy followed by radioiodine ablation. Clinical symptoms varied between the two groups, with FTC patients showing more bone and muscle pain, as well as nephrolithiasis, compared to PTC patients.

Key points

The study emphasizes the need for preoperative ultrasound of the thyroid gland in all PHPT patients to detect any concomitant thyroid disease, thereby aiding in determining the best surgical strategy.

This research significantly contributes to understanding the specific clinical features and differences between PTC and FTC in patients with PHPT-associated NMTC, highlighting the importance of comprehensive preoperative evaluation in this context.

In an article from Riss and coll. [12], 1065 patients who underwent consecutive surgery for primary hyperparathyroidism were analyzed.

Patient analysis and thyroid malignancies

Concomitant thyroid surgery was performed for 502 patients (47.1%), and in 86 patients (8.1%) thyroid malignancy was incidentally detected. Concerning the parathyroid pathology, the majority of patients had single-gland disease, and the initial surgery was successful in curing a high percentage of patients. However, some patients required re-exploration due to persistent disease. RLN palsy rates were comparatively low, but they varied based on the surgical procedure performed, with higher risks associated with more extensive procedures like BNE and thyroid surgeries. The majority of thyroid malignancies were papillary thyroid carcinomas, with a significant number being papillary microcarcinomas.

Key points

The study underscores the need for careful consideration when dealing with these incidental findings. Despite the increased risks for temporary RLN palsy and hypoparathyroidism following concomitant thyroid and parathyroid surgeries, the study highlighted the significance of performing these surgeries, especially in specialized centers in an endemic goiter region. It suggested that the benefits of identifying incidental thyroid malignancies might outweigh the risks associated with the surgeries.

Even Yazici and coll. [13] explored the incidental discovery of PTC in PHPT.

Prevalence of PTC in PHPT

The prevalence of PTC was 37.7% in PHPT patients.

Key points

The study emphasized the need for thorough preoperative assessments. Discovering PTC complicates pHPT treatment decisions, although most found cancers are papillary microcarcinomas, often not requiring additional surgery. The study addresses the challenge of missed thyroid pathology during pHPT surgeries and highlights the importance of evaluating the thyroid to avoid reoperations.

In a study involving 155 patients, Xue and coll. (2016) [14] explored the relationship between PHPT and nonmedullary thyroid carcinoma (NMTC) in a Chinese population. The patients were studied between 2009 and 2014.

Prevalence of PTC in PHPT

NMTC prevalence reached 7.7%, notably higher than in the general population, although lower than the association highlighted in previously reported studies. Interestingly, lower preoperative calcium levels correlated with NMTC presence in PHPT patients with thyroid nodules, suggesting a risk factor.

Key points

The research stresses thorough preoperative thyroid assessments for PHPT patients, which are vital due to evolving minimally invasive surgeries potentially overlooking synchronous thyroid abnormalities.

A study involving 73 patients with concurrent PHPT and thyroid nodules was carried out at our institution [15]. These patients underwent total thyroidectomy and parathyroidectomy.

Diagnostics

Preoperative evaluations of PHPT included ultrasound (US) and MIBI scans, with results aligned with the surgical report. The study found no significant difference between US or MIBI compared to the surgical report. A complementarity between the two investigations was found.

Prevalence of Thyroid Carcinoma

Notably, 32.9% of patients had thyroid carcinoma.

Key points

The study emphasized the necessity of combined US and MIBI in initial assessments and suggesting a more aggressive approach due to the high prevalence of thyroid carcinoma in this cohort.

The study of Çetin (2019) [2] analyzed 275 patients with PHPT who underwent surgery (January 2014 and December 2017).

Prevalence and demographics

Among them, 31 patients were also diagnosed with PTC. Demographic and pathology data of the 31 patients with both PHPT and PTC were compared with 186 patients who underwent thyroidectomy and were diagnosed solely with PTC within the same time frame. The occurrence of PHPT alongside PTC was 11.3% among the patients studied.

The median ages differed significantly among the PHPT, PHPT + PTC, and PTC groups: 55, 57, and 50 years old, respectively (*p* < 0.001).

Tumor characteristics

Tumor size was notably smaller in the PHPT + PTC group, with a median of 7 mm (range 0.5–25 mm) compared to 15 mm (range 1–100 mm) in the PTC-only group. Moreover, microcarcinomas were more frequent in the pHPT + PTC group (*p* < 0.001). Compared to the isolated PTC group, higher rates of tumor capsule invasion and multicentricity in the PHPT + PTC group were found (*p* = 0.02, *p* = 0.04, respectively).

Key points

The smaller tumor size in the PHPT + PTC group might indicate a potential overdiagnosis of PTC due to pHPT. However, the presence of higher rates of tumor capsule invasion and multicentricity in this group suggests a possible associative etiology with more aggressive PTC.

The study of Shen and coll. [16] investigated seven cases of PHPT and PTC concurrently.

Key points

Emphasizing early detection, this study detailed patients’ initial presentation for thyroid nodule checkups, noting ultrasound’s pivotal role in identifying suspected parathyroid lesions. Despite the absence of radiation exposure history, ultrasound successfully detected hypoechoic parathyroid nodules and thyroid lesions. The study compared imaging techniques, favoring ultrasound over 99mTc-MIBI and cervical CT for lesion identification. Surgical interventions, guided by preoperative assessments, led to thyroid surgeries alongside parathyroidectomy. Discussion noted the rarity of this coexistence and unresolved mechanisms. While lacking specific guidelines, the study advocated comprehensive preoperative ultrasound for accurate diagnoses, underscoring its role in refining surgical approaches.

Addressing the role of PTH in thyroid cancer development, this study suggests PTH may not directly contribute. It emphasizes the need for investigating coexistent thyroid nodules in parathyroid patients, which potentially mask underlying malignancies.

A study from Tsai and coll. [17] evaluated prospectively collected data spanning approximately 33 years from a cohort of patients. This cohort initially comprised 2537 individuals with papillary thyroid carcinoma (PTC) and was divided into two groups: one with patients affected by hyperparathyroidism (HPT) and one without. From these groups, two balanced sets were derived using propensity score ratio matching.

Key points

The authors found that PTC coinciding with HPT often exhibits a slow-progressing behavior. Some patients may experience cancer recurrence during a five-year follow-up period. Vigilant and extended surveillance is advisable, particularly for high-risk individuals. Caregivers should take into account cardiovascular risks, especially in cases of severe hyperparathyroidism upon diagnosis.

Liu and coll. (2021) [18] Showed the higher prevalence of thyroid nodules in asymptomatic PHPT patients and the occurrence of PTC in a considerable percentage of these patients. The study also observed differences in the pathological features of PTC between the two groups, particularly a higher rate of microscopic extrathyroidal invasion in the concomitant PTC among asymptomatic PHPT patients.

The article suggests that while asymptomatic PHPT patients might exhibit milder abnormalities in laboratory tests, they might have a higher rate of invasive PTC. Therefore, it emphasizes the importance of considering concurrent thyroid disease during parathyroidectomy in PHPT patients, particularly in cases with suspicious thyroid nodules.

A study from Li and coll. (2021) [19] involved 318 PHPT patients, analyzing thyroid nodules and cancer prevalence.

Prevalence and characteristics of thyroid nodules and malignancies

Results showed a 33% presence of thyroid nodules and an 8.2% incidence of thyroid cancer in these patients. The study aimed to identify malignancy risk factors in PHPT patients with thyroid nodules, examining patient demographics, comorbidities, lab results, and tissue pathology.

Clinical and biochemical profiles, including age, gender, and various blood analyses, were detailed. Histopathological analyses of parathyroid and thyroid tissues highlighted cancer characteristics such as size, histology, bilaterality, multifocality, and lymph node spread.

Correlations of PTH values with cancer

Lower serum PTH levels correlated significantly with thyroid cancer presence, indicating a potential risk factor. Of note, patients with lower PTH levels exhibited larger tumors and multifocality.

Key points

The article stressed the need for deeper exploration into PTH’s role in thyroid cancer risk in PHPT patients. It emphasized the absence of definitive guidelines for managing concurrent PHPT and thyroid nodules, urging further research to guide clinical decisions.

The study of Hu and coll. (2023) [20] encompassed 99 parathyroid adenoma (PA) patients, 22 of whom had concurrent PTC.

Demographics, PHPT clinics and PTC characteristics

Among PA patients, males had higher preoperative PTH and blood calcium levels, and fewer were asymptomatic postoperatively. In the PA + PTC group, preoperative PTH, blood calcium, alkaline phosphatase (ALP), and postoperative PTH levels were lower than in the PA group. The asymptomatic rate was higher in the PA + PTC group. Notably, PA + PTC did not influence PA progression or exacerbate PTC aggressiveness compared to PTC alone. However, PA + PTC demonstrated a lower lymph node metastasis rate than PTC alone. PA characteristics included occurrence across all ages, more prevalence in women but more severity in men, and a tendency to locate in the lower pole of the gland. The coexistence of PTC and PA might enable early disease detection.

Key points

With 22.2% of PA patients presenting PTC, surgeons should diligently monitor thyroid disease to avert reoperation. The study’s findings suggest that while PA and PTC coexistence does not worsen either condition, it may aid in earlier disease diagnosis.

A recent study from Gucek Aciyanli (2022) [21] aimed to explore factors influencing the type and extent of surgery in patients with primary hyperparathyroidism (PHPT) and concomitant thyroid disease, particularly thyroid cancer. Over a 6-year period, records of patients undergoing parathyroidectomy for PHPT were reviewed.

Prevalence of thyroid disorders and malignancy in PHPT

Out of 284 patients, 158 (55.6%) had concomitant thyroid disease, and 84 (29.6%) underwent both parathyroidectomy and thyroidectomy. Among these, 29 (10.2%) were diagnosed with thyroid carcinoma.

Surgery complications and risk factors for malignancy

Comparing two groups—benign thyroid disease (Group 1) and malignant thyroid disease (Group 2)—based on histopathological examination, it was found that total thyroidectomy and complication rates were higher in Group 2. Despite preoperative fine-needle aspiration biopsy being obtained in 58.3% of patients, it only identified 26.3% of confirmed thyroid carcinoma cases. Notably, higher preoperative serum phosphorus levels were observed in Group 1, but no parameter emerged as an independent risk factor for thyroid malignancy in multivariate analysis.

Key points

In conclusion, no definitive parameter may allow the prediction of concurrent thyroid carcinoma in PHPT patients. Even with a benign biopsy, thyroid malignancy cannot be ruled out, potentially leading to undiagnosed thyroid carcinoma. In particular in this context papillary microcarcinoma is very frequent.

Lastly, some case reports report anecdotal associations and features such as thyroid and parathyroid carcinoma (intrathyroidal), parathyroid adenoma, papillary thyroid carcinoma and thyroid adenoma in a pregnant woman, medullary thyroid carcinoma, PTC and PHPT due to PA, the Hobnail variant of PTC and PHPT, follicular thyroid carcinoma, PTC and PHPT, functioning parathyroid cysts, and PHPT [22,23,24,25,26,27].

The results obtained in this narrative review are therefore derived from a total of 7941 patients included in the reviewed studies, plus 6 case reports, for an overall total of 7947 patients.

Are there differences between PHPT and SHPT?

Only three articles have examined the SHPT–PTC association [17,27,28], one of which [18] does not make a precise distinction in this regard, assuming that the two forms have similar prevalence rates. To be fair, this study has the limitation of looking for the association of HPT from the PTC.

In a comprehensive study involving 217 patients suffering from primary (140 patients) and secondary (77 patients) HPT, Preda and coll. (2019) [27] examined the correlation between PHPT and SHPT and thyroid cancer. Among the patients with SHPT, 31 (40.3%) had both thyroid and parathyroid surgery, revealing 9 cases of papillary thyroid carcinoma (PTC), accounting for 11.7% of SHPT cases and 25.7% of those who had both surgeries. Adenomatous/colloid goiter was the most common thyroid issue among these patients (51.7%). Assessing patients undergoing parathyroidectomy and thyroid surgery, it uncovers the following key points:

PTC prevalence and characteristics: Notably, both PHPT and SHPT cases revealed a significant occurrence of papillary thyroid cancer (PTC), accounting for 13.6% and 11.7%, respectively. Variations in tumor size and features were observed; larger PTCs were prevalent in PHPT, while SHPT exhibited micropapillary tumors predominantly.

Surgical Approach: PHPT leaned towards minimally invasive parathyroidectomy, while SHPT required subtotal parathyroidectomy due to gland hyperplasia.

Gender and Age: PHPT favored females in their fifth to sixth decades, while SHPT occurred across genders, albeit at a slightly younger age.

PTH Levels: SHPT patients displayed notably higher preoperative PTH levels compared to PHPT cases.

Jeong (2020) [3] evaluated patients who underwent parathyroidectomy between January 2009 and December 2019 in two medical centers. Among 279 participants, 154 had PHPT, and 125 had SHPT. Comparisons were made with 98 patients diagnosed with classical PTC after thyroidectomy during the same period.

PTC prevalence and characteristics

Concurrent PTC occurred in 14 (9.1%) participants with PHPT and 9 (7.2%) with SHPT.

Most PTCs were microcarcinomas in both groups. Lower vitamin D levels were observed in pHPT + PTC (13.0 ± 3.7 ng/mL) compared to PHPT only (18.5 ± 10.4 ng/mL; *p* = 0.01). A similar trend was seen in SHPT + PTC (12.3 ± 5.6 ng/mL) versus SHPT only (18.0 ± 10.2 ng/mL; *p* = 0.12). The lymph node ratio was higher in the concomitant PTC group compared to classical PTC (*p* = 0.00).

Key points

A significant prevalence of concurrent PTC was found in PHPT and SHPT patients, mostly as microcarcinomas with more aggressive features. Emphasizing the importance of identifying simultaneous malignancies during preoperative parathyroidectomy evaluation is warranted.

Our review found only one article exclusively addressing SHPT: Ma and coll. (2021) [29] evaluated the occurrence of PTC in patients with SHPT. The study aimed to assess whether SHPT is associated with more aggressive PTC in terms of tumor characteristics and prognosis.

PTC prevalence and characteristics

The research analyzed 531 patients with SHPT, among whom 34 were found to have PTC. Comparing these patients with a control group of 136 PTC patients without SHPT, they found that the PTC in the SHPT group tended to be smaller and more often classified as papillary thyroid microcarcinomas (PTMCs). However, they observed no significant differences in terms of tumor multicentricity, bilaterality, extrathyroidal extension, lymph node metastasis rate, or prognostic staging between the PTC in the SHPT group and the control PTC group.

Key points

The study suggested that while SHPT might increase the prevalence of PTC, the characteristics of PTC in SHPT patients were more frequently occult thyroid carcinomas, showing no significant differences in aggressiveness compared to PTC in the general population. The data also indicated a higher incidence of PTC in surgically treated SHPT patients.

The researchers recommended thorough preoperative examinations and careful surgery to avoid missing the coexistence of PTC in patients with SHPT.

## 4. Discussion

HPT and PTC are common diseases [30,31]. HPT needs to be distinguished from other conditions, such as familial hypocalciuric hypercalcemia [32]. The diagnosis of PHPT involves initial investigations (scintigraphy, ultrasound), while second-level tests like PET-choline and 4D-CT are emerging for their diagnostic accuracy [33,34]. Intraoperative PTH is an important method for early confirmation of curing and to prevent persistence or recurrence [35,36]. For PTC and generally all thyroid tumors, ultrasound and FNAB are crucial for diagnosis [37,38].

The collected studies, summarized in Table 1 and Table 2, converge on understanding the relationship between primary PHPT and thyroid conditions, emphasizing the prevalence of thyroid nodules and thyroid cancer in PHPT patients. Notable similarities across studies include the incidence of PTC in PHPT patients, ranging from 2.8% to 47.1%, with some studies highlighting a significant coexistence of PTC with PHPT [11,12,15]. Intriguingly, findings highlight lower serum PTH levels correlating with thyroid cancer in PHPT patients, suggesting a potential risk factor [20]. Variations in tumor characteristics and surgical approaches were observed, with studies indicating a higher incidence of PTC in females, varying tumor sizes, and differences in PTH levels between PHPT and secondary hyperparathyroidism (SHPT) cases. While studies highlighted the importance of preoperative assessments and the benefits of identifying incidental thyroid malignancies, they stressed the lack of definitive guidelines for managing concurrent PHPT and thyroid conditions, advocating for comprehensive evaluations to refine surgical strategies. Some reports also note rare coincidences like intrathyroidal parathyroid carcinoma, PTC and PHPT in pregnancy, and functioning parathyroid cysts with PHPT, shedding light on unique associations between thyroid and parathyroid disorders.

Summary

In the light of the literature review carried out, the essential points that emerge from this review are as follows:

Association confirmation: The collection of studies reinforces the established association between hyperparathyroidism (HPT) and thyroid carcinoma (TC), emphasizing the prevalence of thyroid nodules and thyroid cancer in patients with primary hyperparathyroidism (PHPT).

Incidence Variation: Studies showcase a wide range of incidence rates for TC in PHPT patients, from 2.6% [13] to 32.9% [15]. It should be noted that in the study carried out at our institution [15], an important selection bias could be the initial recruitment of the two conditions (PHPT and thyroid nodules) in coexistence. Similar consideration could be had concerning the study from Hu [22]. Therefore, it is likely that a high number of thyroid carcinomas were found in this context. In any case, this variability suggests potential differences in patient cohorts, geographical influences, or diagnostic approaches.

Risk Factors: Lower serum parathyroid hormone (PTH) levels seem to correlate with the presence of thyroid cancer in PHPT patients, indicating a potential risk factor for the coexistence of these conditions. In particular, two studies (Preda [27] and Liu [18]) highlight this difference. The articles considered do not seem to highlight in detail what role radiation exposure might play, although it is known that this risk factor affects both PHPT and PTC [2,5,15].

Gender and Tumor Characteristics: Variations in tumor characteristics and surgical approaches were observed, with differing incidences among genders and variations in tumor sizes, highlighting potential differences in disease behavior between PHPT and secondary hyperparathyroidism (SHPT) cases. An aspect that stands out is the high frequency of PTmC within the general context of identified TCs, predominantly papillary. When it is not possible to ascertain the prevalence of PTmC among the various carcinomas, the average tumor size is consistently confined within 1.4–1.6 cm [11,12,13,14,15,16,17,18,19,20,21,22,23].

Regional differences: The majority of the studies (eight) came from the Far East, while seven were carried out in Europe. We found no studies from the United States. The geographical data do not seem to lead to any different conclusions.

Differences between PHPT and SHPT: PTC in SHPT tended to be smaller (most frequently PTmCs), displaying no significant differences in aggressiveness compared to the general population. However, it must be considered that PHPT predominantly affected females in their fifth to sixth decades, while SHPT occurred across genders at a slightly younger age range.

Diagnostic Challenges: The studies underscore the diagnostic challenges associated with identifying incidental thyroid malignancies during PHPT surgeries. This emphasizes the need for more comprehensive preoperative evaluations to refine surgical strategies and avoid missed diagnoses.

Need for Guidelines: The lack of definitive guidelines for managing concurrent PHPT and TC is a significant concern highlighted across multiple studies. This gap in guidance underscores the necessity of further research to establish clear protocols for managing these coexisting conditions.

*Unique Associations*: Some reports highlighted rare associations between thyroid and parathyroid disorders, shedding light on unique coincidences that require deeper exploration and understanding.

*Clinical Implications*: Understanding the interrelationship between HPT and TC is essential for refining diagnostic and therapeutic approaches. Additionally, it is crucial to develop guidelines to optimize the management of patients presenting with both conditions.

*Calcium levels and NMTC*

Confirmations have indicated a higher likelihood among patients with thyroid nodules, particularly for slightly elevated values. Previous research [14] demonstrated that within patients with primary hyperparathyroidism (PHPT), individuals with NMTC exhibited lower preoperative serum calcium levels compared to those with benign thyroid nodules. Our study further establishes a significant inverse correlation between preoperative serum calcium levels and the presence of NMTC among PHPT patients. Our findings suggest that serum calcium levels corrected for albumin below 2.67 mmol/L could effectively differentiate PHPT patients with NMTC from those with benign thyroid nodules. The findings hint that PHPT might increase the risk of thyroid nodule malignancy, and lower serum calcium levels could potentially predict the presence of NMTC in PHPT patients with thyroid nodules.

*Potential limitation of the study*

The contribution of clinical cases to this review was not substantial, except to bring to attention conditions that might be encountered in clinical practice on other occasions (coexistence of more aggressive neoplastic forms, unusual forms of parathyroid lesion, association of different types of thyroid carcinoma). Their inclusion is therefore aimed at describing those events that might require specific attention.

Narrowing the search to the decade of 2013–2023 may have limited the information gained from this review. However, we believe that the data obtained from over 7900 patients in total involved in the individual papers provide sufficient background for understanding the topic. Furthermore, extending the focus further back seemed unnecessary, as it would likely yield redundant data. This choice would appear to be a limitation of this study, but incorporating useful diagnostic insights from earlier research was challenging due to recent advancements in this field.

## 5. Conclusions

In essence, while confirming the association between HPT and TC, these studies reveal a need for standardized diagnostic protocols, clearer risk factor delineation, and comprehensive guidelines for managing these concurrent conditions, ensuring optimized patient care and outcomes.

## Figures and Tables

**Table 1:** Studies on association between thyroid carcinoma and hyperparathyroidism.

First Author	Publication Date and Country	Study Type	Enrolled Patients	Type of Association Highlighted	Notes
Lehwald [11]	2013 Germany	Retrospective	1464	Coexistence of NMTC and PHPT; prevalence 2.8%.	Predominant NMTC: PTC; tumor characteristic variations.
Riss [12]	2015 Germany	Retrospective	1065	Association of pHPT and thyroid malignancy	Higher PTC prevalence; some cases required reoperation.
Yazici [13]	2015 Turkey	Retrospective	228	37.7%: concurrent thyroid disease; 6.9% malignancies	Low rate of malignancies seems coincidental.
Xue [14]	2016 China	Retrospective	155	Association of NMTC and PHPT; prevalence 7.7%.	Correlation of NMTC with low preoperative calcium levels.
Scerrino [15]	2016 Italy	Retrospective	73	Association of PHPT and thyroid malignancy	Combined US and MIBI in initial assessments, suggesting a more aggressive approach.
Çetin [2]	2019 Turkey	Retrospective	275	Smaller in the PHPT + PTC group, but signs of higher aggressiveness	PHPT leads to overdiagnosis of PTC?
Shen [16]	2019 China	Observational	112	PTC in PHPT: 6.25%	Pivotal role of ultrasound over 99mTC-MIBI.
Tsai [17]	2020 Taiwan	Case-control Prospective	2537	PTC in HPT with slow progression; recurrence possible	Recommendations for extended surveillance.
Liu [18]	2021 China	Observational	304	PTC more frequent in asymptomatic pHPT	FNA is mandatory, especially in asymptomatic cases.
Li [19]	2021 China	Observational	318	Prevalence of thyroid nodules and carcinoma; incidence 8.2%	Lower PTH levels correlated with thyroid carcinoma presence.
Hu [20]	2023 China	Observational	99	PA and PTC coexistence; 22.2% PA had PTC.	PA and PTC coexistence for early diagnosis.
Gucek Aciyanli [21]	2022 Turkey	Retrospective	284	Concomitant thyroid disease in 55.6%; malignancy in 34.5%.	Careful evaluation in the presence of both HPT and thyroid disease.
Preda [28]	2019 Romania	Observational	217	Significant PTC incidence in PHPT and SHPT	Variations in tumor characteristics and surgical approaches.
Jeong [3]	2020 Korea	Retrospective Comparative	279	PTC: 9,1% in PHPT and 7.2% in SHPT.	Mostly microcarcinomas; more aggressive features
Ma [29]	2021 China	Retrospective, Matched-control	531	SHPT-associated PTC	SHPT linked to PTC prevalence but similar aggressiveness.

**Table 2:** Case reports on coexistence of thyroid carcinoma and hyperparathyroidism.

First Author	Publication Date and Country	Case Report Type	Association Highlighted
De Falco [22]	2021 Italy	Intrathyroidal parathyroid and thyroid carcinoma	Unique association between parathyroid and thyroid carcinoma
Li [23]	2020 China	PTC and PHPT in pregnancy	Rare simultaneous occurrence in pregnancy
Anagnostis [24]	2018 Greece	Functioning parathyroid cyst and PHPT	Association of parathyroid cyst and PTC
Wang [25]	2020 China	Medullary thyroid carcinoma, PTC, and PHPT due to PA	Multiple carcinoma types coexisting with PA
Dai [26]	2019 China	Follicular thyroid carcinoma, PTC, and PHPT	Simultaneous occurrence of different thyroid carcinomas with PHPT
Al-Yairi [27]	2020 Qatar	Hobnail variant of PTC and PHPT	Unique subtype of PTC with PHPT association
